# The Eating Cure

**Published:** 1870-10

**Authors:** 


### THE EATING CURE.

The book on "Health by Good Living" has met with a favorable reception, about a thousand copies having been sold a week since its first issue, some three months ago. The general positions taken are, that—

1. All ordinary diseases arise from bad blood.

2. This bad blood must in all cases be removed from the system before health can be restored.

3. How this can be done uniformly without medicine and without any expenditure of money.

4. Bad blood must be replaced by good and pure blood as a result of eating good food, well prepared, with a vigorous appetite.

5. How this can be always secured without cost—the appetite, not the food.

6. The necessity of a vigorous, healthy digestion, and the way to obtain it.

7. Cases showing that these things have been done, and can be done to an indefinite extent.

8. The mode of cure is safe, radical and lasting.

9. How to keep well, and how to avoid ordinary diseases.

The author greatly desires that the book should be placed in the hands of the young, as a means of showing them in a plain way how to live so as to avoid disease; what habits they should abandon in reference to eating, drinking, exercise, sleeping, and the like. It does not tell them simply to do this or avoid that, but gives the reason for so doing, in such a manner, that it cannot fail to impress the memory, so that it cannot be forgotten as long as life lasts; nor can the judgment fail to be convinced, because the things stated are fixed and universally conceded physiological laws.

Reading the book can only inform the intellect—the body must be benefitted by the practice of the suggestions made. One thing has gratified the author very much—the confirmations of the things stated in the book, as curing certain maladies, by persons who had successfully practiced those things many years ago, before the book was ever thought of, having been led thereto by their own observation, judgment and deductions. And as some of the subjects treated are of personal interest to all—such as what to eat, when to eat, how much to eat, etc., scarcely any one can read it without more or less profit. A correspondent writes:

"A LADY

stepped into our office the other day, and said, 'I saw your notice of Dr. Hall's book on "Health by Good Living," and if it is as good as you say it is, I want it.' A few days after she called and said,

'I AM DELIGHTED WITH IT; it is just what I wanted; I have urged several of my lady friends to get it, and they have bought several copies already.' And," continues our correspondent, "if every lady was as much interested in commending the good books they read, how much greater power for good would a healthful, useful literature exert!"

Parents come to consult a physician sometimes for their opinion, and would give half their fortunes if health could be given back, when a dollar or two expended a few years earlier in such a book as this, or another called "Health and Disease," as affected by constipation, and its unmedicinal cure, or for a few numbers of *Hall's Journal of Health*, all this sickness might have been easily prevented.

"THE NIGHT-TIME OF LIFE."

We have written another book on "Sleep," or the "Hygiene of the Night," showing what an important bearing that portion of our lives spent between the closing of our chamber doors at night, and opening them in the morning, has on the health and happiness of individuals and families. No book has ever been written on that subject before, and hence it is

A NEW THING UNDER THE SUN.

One third of our entire existence is spent in our chambers, and whether we breathe a bad or good atmosphere all that time, must have an important influence on health; and in order to impress this fact upon the young, some of the most terrible narrations are recorded that human pens have ever written, showing that breathing a bad air for an hour may engender fatal diseases.

Another subject is treated, of incalculable importance—that of getting abundant, healthful sleep; in fact, it is a universal injunction,

"GET ALL THE SLEEP YOU CAN,"

applicable to children, to mothers while nursing, to brain-workers and day laborers. Rules are given how to get sound sleep, and how much to sleep.

But other subjects are treated; among these are,

THE BAD HABITS OF YOUTH, and certain vicious practices at night—what they lead to, and how their destructive effects can be rectified. Then again there is a certain class of books about

MARRIAGE AND MANHOOD; about physiology, loss of abilities, and many other abominations, with warnings against the purchase of such, as written by unprincipled men, giving their stolen certificates and forged recommendations, for the purpose of inspiring confidence, of imposing on the unwary and alarming the timid, as a means of fleecing them of their money, by all sorts of lying representations and plausible statements. The common steps are these :

### FIRST STEP.

There is no doubt whatever you can be cured. Have had many cases like yours, and never failed.

### SECOND STEP.

One "course of medicine" having been taken, another is advised, "as it is a more stubborn case than was expected. Stronger remedies must be applied ; these will cost a little more than the first, but they have never been known to fail."

### THIRD STEP.

Yours proves an unusually aggravated case. And as you seem to be out of money, and it would cost a good deal to go through a "third course," you must either do that, or

### "GET MARRIED."

"But, my dear sir, nobody would have me ; besides, I have no money." Then, as a means of getting rid of moneyless patients, the way is paved and made easy towards alliances, which end in perdition here and hereafter. All this is horrible to think of, and yet respectable newspapers print the advertisements of these men.

### NURSING MOTHERS.

Many mothers are worn out by their infant children preventing their needed sleep at night ; instructions are given on this most important subject, showing that by proper management the great annoyance of the night, of

### CRYING BABIES, may be obviated, to the health of both mother and child. Among other subjects discussed, is that of

### SLEEPING TOGETHER.

The book advocates the idea that all who can should have single beds and separate rooms, as essential to comfort, healthfulness and the real enjoyment of sound, refreshing sleep ; that the young suffer from sleeping with the old—the well with the sick ; and that persons of different temperaments or different electrical states cannot otherwise than suffer from sleeping together.

### BUSINESS AND SLEEP.

The necessity of sound sleep, as a qualification for transacting all business successfully, is insisted upon. Occasion is taken for impressing upon the reader's attention, by analogies, the absolute need of having metes and bounds to eating and drinking, and all the appetites of our nature, and what are proper restrictions in the several cases ; the value of regularities ; the influence of late eating and drinking on the soundness of sleep. And then again are discussed those various questions in this connection which influence the peace and happiness of whole families ; how from slight causes, from want of consideration, from want of certain sympathies, from want of reflection, fire-brands are sometimes cast which drive a man from his own fireside, to take refuge in the tavern, the corner grocery, the club-house and the brothel. For want of a single moment's reflection, whole families are often made miserable, happy homes are clouded, and social, domestic and pecuniary ruin follow on apace.

Another wide-reaching subject is treated, showing that with the proper conduct of the night-time of life is connected the subject of congenital diseases and imperfections ; that is, it shows why some are born with diseased constitutions, imperfect senses, deficient intellect, etc. Surely every parent should be intensely interested in knowing that these calamities can be avoided always by studying and practicing

"THE HYGIENE OF THE NIGHT."

